# Acquired haemophilia as a paraneoplastic manifestation of pancreatic cancer

**DOI:** 10.3332/ecancer.2020.1053

**Published:** 2020-06-04

**Authors:** Abeer Arain, Ibrahim N Muhsen, Maen Abdelrahim

**Affiliations:** 1 Houston Methodist Cancer Center, Houston Methodist Hospital, Houston, TX 77030, USA; 2 Department of Medicine, Houston Methodist Hospital, Houston, TX 77030, USA; 3 Cockrell Center for Advanced Therapeutic—Phase I program, The Methodist Hospital Research Institute, Houston, TX 77030, USA; 4 Weill Cornell Medical College, Institute of Academic Medicine, Houston, TX 77030, USA; a https://orcid.org/0000-0001-6295-4318

**Keywords:** acquired haemophilia, pancreatic cancer, inhibitor treatment, paraneoplastic phenomenon

## Abstract

Acquired haemophilia is a severe haematological disorder characterised by the presence of anti-factor VIII antibodies. Although rare, it can lead to serious bleeding complications. Acquired haemophilia can be seen in patients with malignancies as a paraneoplastic phenomenon. This is a case of a 52-year-old patient who presented with haematuria and retroperitoneal bleeding soon after being diagnosed with pancreatic adenocarcinoma and subsequently was found to have acquired haemophilia. The treatment of underlying malignancy with chemotherapy may accelerate the eradication of anti-factor VIII antibodies.

## Introduction

Acquired haemophilia (AH), although rare, has been associated with potentially serious complications, such as life-threatening haemorrhages [1]. The incidence is about 0.2 to 1.48 per 100,000 population per year [2]. The mortality is between 8% and 44%, much higher compared with inherited haemophilia [2]. Unlike the traditional haemophilia, AH is not secondary to a familial history [2]. It occurs from the lack of immune tolerance leading to the formation of autoantibodies (IgG oligoclonal or restricted polyclonal) directed against the functional epitope factor of factor VIII (FVIII) [3]. This results in increased clearance or neutralisation of FVIII [4]. Bleeding in AH is mostly in the form of mucosal, or deep subcutaneous and less commonly with haemarthrosis which occurs more in inherited haemophilia [4].

AH commonly presents in a biphasic age distribution. For instance, it occurs in women in the younger years mostly during the post-partum period due to the formation of antibodies. Among older patients, there is increased male predominance, between the ages of 68–80 years [5]. Most of the time, approximately 50%, the antibody formation is idiopathic in nature [6]. It is also can be associated with autoimmune disease, such as Grave’s disease, as well as with hepatitis B or C, diabetes mellitus and pulmonary diseases. About 10% of patients with AH have an underlying malignancy [6].

We present a case of a 52-year-old man who presented with fatigue, weight loss and abdominal pain, was found to have locally advanced pancreatic cancer, and soon developed severe refractory anaemia and was diagnosed with AH in the setting of malignancy. The treatment of the cancer with chemotherapy leads to the recovery of his factor VIII levels.

## Case presentation

Our patient was a 52-year-old man who initially presented to the hospital with abdominal pain, weight loss and fatigue. He had no significant past medical or surgical history. The patient reported heavy alcohol and tobacco use for >10 years. He was initially treated at a hospital in a rural part of Texas for pancreatitis and was discharged home. The patient presented a few weeks later with progressive jaundice and weight loss, diarrhoea, poor appetite and severe nausea.

**Figure 1.**
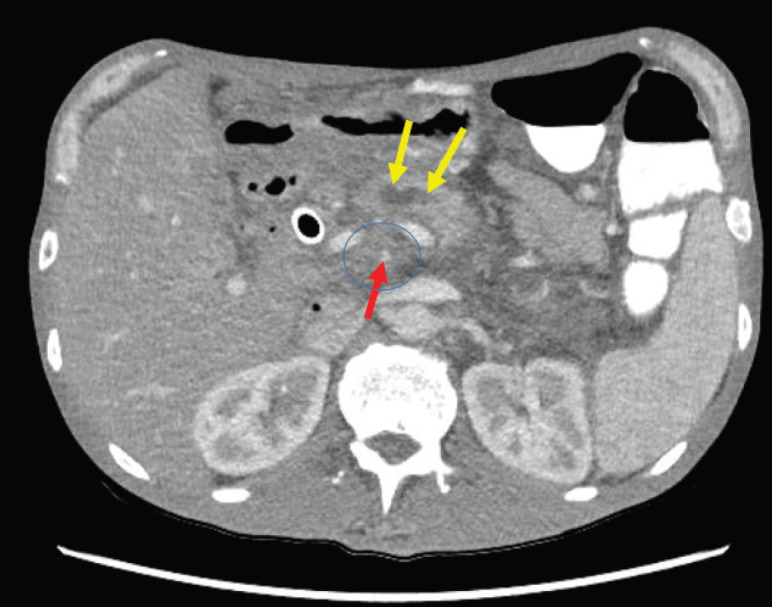
CT Abdomen showed 3.5 x 2.5 cm primary tumour in pancreatic head/uncinate process mass with complete encasement of superior mesenteric artery (SMA)/superior mesenteric vein (SMV). No evidence of any regional lymphadenopathy or liver metastasis. The figure illustrates the approximate extent of pancreatic mass (circled), pancreatic duct dilatation (yellow arrow) and the narrow superior mesenteric artery (red arrow).

A CT scan of the abdomen reported a newly-found pancreatic mass in the pancreatic head/uncinated process (Figure 1), about 3.5 × 2.5 cm in size and encasing the superior mesenteric artery and occluding the superior mesenteric vein near the splenoportal confluence. Biliary ductal dilatation was also reported. The patient underwent endoscopic retrograde cholangiopancreatography (ERCP) and a metal stent was placed in the common bile duct. Post-ERCP, patient was discharged home to follow-up in the oncology clinic. The biopsy of the pancreatic mass reported pancreatic adenocarcinoma, the patient was found to have locally advanced disease with no distant metastases. CA19-9 was 142 U/mL (ref 0–35 U/mL).

**Figure 2.**
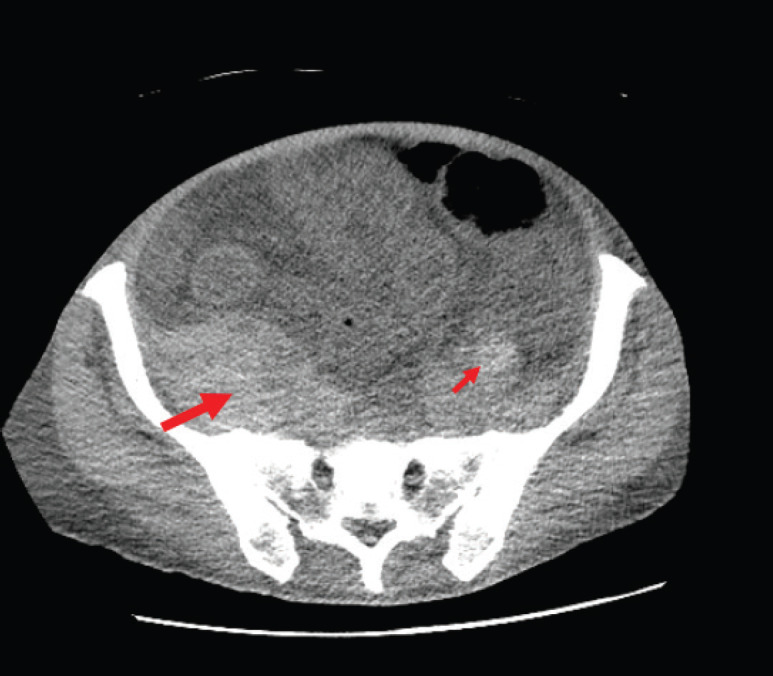
CT Abdomen showing severe anasarca and retroperitoneal haemorrhage. The figure shows the bilateral retroperitoneal haemorrhages both right sided (thick arrow) and left sided (thin arrow). It illustrates the asymmetry with right side retroperitoneal haemorrhage being larger in size.

The patient, however, presented to the ER with dyspnoea, fatigue, nausea, generalised abdominal pain and haematuria. Upon imaging, he was found to have a newly-developed retroperitoneal bleed (Figure 2). Initial haematology labs reported severe anaemia with Hb level of 4.5 g/dL (ref 14–18 g/dL), mild leukocytosis of 12.17 k/μL (ref 4.5–11.0 k/μL), and normal platelet count of 202 k/μL. Coagulation studies showed elevated prothrombin time (PT) of 17.9 seconds (ref 12–15 seconds), elevated partial thromboplastin time (PTT) of 104.9 seconds (ref 23–36.0 sec) and international normalised ratio of 1.4. Chemistry results showed acute renal injury with creatinine of 3.6 mg/dL (ref 0.7–1.2 mg/dL and patient’s baseline of 0.9 mg/dL). Patient was initially managed with blood transfusions, intravenous (IV) fluids, IV vitamin K given active haematuria. Mixing studies were ordered to further evaluation of the deranged coagulation panel. Peripheral smear was negative for spherocytosis or schistocytosis. A work-up of the patient bleeding yielded no signs of acute haemolysis (or deep venous thrombosis), fibrinogen levels were normal and patient’s coagulation factors studies were unremarkable, except for severe decrease in his factor VIII activity. Mixing studies revealed prolonged PT and PTT with 1:1 mix correction of PT and no correction of PTT and a 14.2 seconds post-incubation period, pointing towards decreased factor VIII activity. These results were suggesting the presence of a factor inhibitor. Bethesda assay titer was sent and results shown high levels of factor VIII inhibitor which was 13.2 (ref < 0.5) with factor VIII of <1%. Plans for chemotherapy were put on hold at that time and patient was started initially on recombinant factor VIIa (rVIIa), and afterwards prednisone and rituximab were added. The recombinant factor VIIa was given at a dose of 70 mcg/kg with decreasing frequency (initially every 2 hours, followed by every 6 hours and then every 12 hours) and was used in the first 2 weeks of admission, guided by the patient’s haemoglobin. Following that, prednisone was started at 1 mg/kg and was also given for the first 2 weeks followed by tapering course. Given the severity of the presentation, cyclophosphamide and rituximab were considered, however, rituximab was favoured given the patient presentation with haematuria and possible cystitis. The patient received 1 dose weekly of rituximab for 3 weeks at a dose of 375 mg/m^2^).

**Figure 3.**
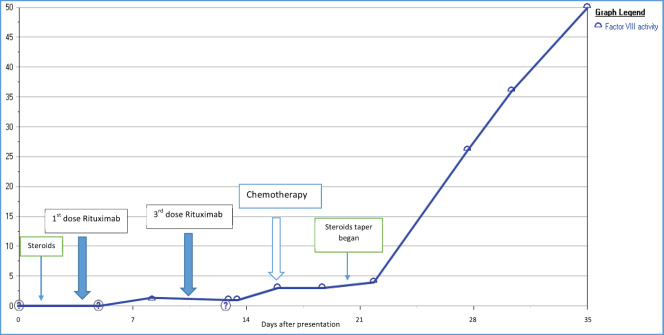
Increasing factor VIII level as chemotherapy was given, also shows timeline of other used immunosuppressive therapies.

The patient’s pancreatic cancer was found to be unresectable. Given his clinical presentation with locally advanced pancreatic cancer and AH, it was decided to start chemotherapy as soon as the patient is stable. Four cycles of dose-adjusted Gemcitabine and nab-Paclitaxel were planned and the first cycle was given while inpatient. Patient had a prolonged hospital stay of 4 weeks and in this time he received a total of 3 doses of Rituximab, and cycle 1 of chemotherapy. Steroids were tapered slowly. Factor VIII level started to increase as the chemotherapy was started (along with Rituximab infusions and high-dose steroids which were also given), and it reached to the value of 50% by the time patient received his second cycle of chemotherapy using the Gemcitabine and nab-Paclitaxel regimen (Figure 3). Later, in his course of treatment, patient unfortunately developed septic shock from Gram-negative bacteraemia and severe respiratory failure that was treated and stabilised, however, later patient and the family wished to change goal of care to hospice.

## Discussion

AH is considered to be associated with high risk of bleeding and increased risk of complications such as frequent haemorrhages [7]. The bleeding usually involved the skin, muscles and soft tissues. Several diseases have been associated with AH in the literature. Malignancies are associated about 10% of the time [6]The spontaneous emergence of factor VIII inhibitor in patients with cancer creates a clinical challenge for the physician and further increases the chances of complications for the patient, such as increasing risk of bleeding [3].

Studies have reported the association of solid organ malignancies, such as gastrointestinal stromal tumours, duodenal or pancreatic cancers with Acquired haemophilia in the form of case reports [8, 9]. Bleeding can be a consequence of chemotherapy induced side-effects, but at the same time it is crucial to think of the other differentials such as factor VIII deficiency, disseminated intravascular coagulation (DIC) or invasion of large vessels by the tumour [3]. The approach to patients with bleeding diathesis starts with a broad differential to avoid missing any possible cause. Although the priority is always the stabilisation of the patient, diagnosing the patient is crucial to be able to start the patient on the correct therapy. Cancer patients are at risk of multiple bleeding disorders, including DIC, bleeding related to anti-coagulants, dysfibrinogemia, etc. Certain cancers are at risk of certain diseases, such as Acquired von Willebrand disease in cases of lymphoma [10]. Additionally, certain patterns of DIC are seen in different types of cancer; for instance, DIC is likely to present with hypercoagulable pattern in solid cancers but with bleeding in haematologic malignancies. Dysfibrinogemia/hypofibrinogenemia is another important differential that happen when there is a qualitative or quantitative decrease in fibrinogen, which can happen secondary to congenital and acquired diseases. However, it is more common in cases of haematologic cancers particularly myeloma [11]. These causes of bleeding diathesis must be taken into consideration and subsequently work-up should be tailored to the exclusion of these etiologies.

Studies in the literature have reported the outcomes of the patients who have malignancy and are also found to have factor VIII deficiency [12]. Sallah *et al* [9] studied a retrospective analysis of 41 patients with cancer and acquired haemophilia. The most common malignancies reported were prostate cancer in solid tumours and chronic lymphocytic leukaemia in haematological malignancies [9]. The study reported that chemotherapy, surgery or hormonal manipulation has led to the disappearance of factor VIII inhibitor [9]. Similar response was observed in our patient, when he received chemotherapy for pancreatic cancer, he had a subsequent increase in his factor VIII levels as shown in Figure 3. The rise of factor VIII and the reduction of the antibody were noticed around the time of cycle 1 when the chemotherapy was administered. Our patient likely benefited from the therapy of AH given, including high-dose steroids, rituximab and rVIIa.

AH should be considered in every patient who comes with an unexplained bleeding episode regardless of the underlying comorbidities [8]. The coagulation profile must be thoroughly checked and upon suspicion, mixing studies and Bethesda assay should be ordered. However, the treatment decisions should be guided by the severity of bleeding and not by Bethesda titers [8]. Different reports in the literature have highlighted the first-line therapy for patient presenting with bleeding secondary to acquired haemophilia. The two main lines of therapy are the haemostatic therapy for the haemorrhage and immunotherapy in order to eradicate the antibodies [8]. At most centres, the first-line therapy usually includes the recombinant factor VII or activated prothrombin complex concentrate is used until the bleeding is controlled [13]. Steroids alone or in combination with cyclophosphamide has also been a recommended first-line therapy. This combination, however, takes a few weeks to show clinical response. Therefore, rituximab along with high-dose steroids is an alternative regimen being used at most centres [3, 8]. For our patient, we opted for the combination regimen and used recombinant factor VII initially along with steroids followed by Rituximab infusions [14, 15]. We also started chemotherapy for pancreatic cancer using gemcitabine and nab-paclitaxel in the same admission. This case highlights the importance of identifying factor VIII inhibitor, or AH, in patients with malignancy and prompt management using the recommended protocols. Interestingly in our patient, the eradication of the antibodies against factor VIII was accelerated when chemotherapy regimen was started for primary malignancy. Our recommendation is to start chemotherapy in such case soon after patient stabilisation to accelerate and augments the benefit of AH-directed therapy and avoid recurrent admissions and interruptions in their primary cancer therapies.

## Conclusions

In this paper, we discussed a case of acquired haemophilia due to pancreatic cancer. Few cases have been reported in the literature. Along with immunosuppressive therapy, this case supports the initiation of chemotherapy in treating acquired haemophilia. Acquired haemophilia is a diagnosis that should be considered in cancer patients presenting with bleeding; however, other causes of coagulopathy should be considered too. Nevertheless, initial efforts should be targeted to the stabilization of patients. More studies are needed to clarify the pathophysiology of antibodies formation in malignancy.

## Conflicts of interest

None of the authors declare any relevant conflicts of interest.

## Funding statement

No funding support was obtained.

## Authorship contributions

AA and MA wrote the first draft of the manuscript. All authors vouch for the accuracy and contents of the manuscript. All authors approved the final version of the draft.
